# Clinical Characteristics, Metabolic Parameters, and Risk Factors for Suicide Attempts Vary with Untreated Major Depressive Disorder Duration

**DOI:** 10.1155/2023/4869276

**Published:** 2023-12-21

**Authors:** P. Tong, Y. H. Shi, Y. Yang, L. P. Dong, L. L. Wu, T. T. Sun, W. Lu, X. Y. Zhang

**Affiliations:** ^1^Department of Clinical Psychology, Northern Jiangsu People's Hospital, Yangzhou 225001, China; ^2^Medical College, Yangzhou University, Yangzhou 225001, China; ^3^Department of Neurology, Danyang People's Hospital, Danyang 212300, China; ^4^CAS Key Laboratory of Mental Health, Institute of Psychology, Chinese Academy of Sciences, Beijing 100101, China; ^5^Department of Psychology, University of Chinese Academy of Sciences, Beijing 100101, China

## Abstract

Suicidal attempts (SAs) are common in major depressive disorder (MDD). However, only few studies have so far assessed how risk factors for SAs in patients with MDD might be related to the duration of untreated illness (DUI). We interviewed 1,718 drug-naive outpatients with MDD with first-episode SAs and divided them into groups that had and had not attempted suicide. DUI was used as an additional grouping criterion. The patients (20.14%; 346/1718) who had a history of SAs were older and had a longer DUI; lower educational level (middle school-educated only); more psychotic symptoms; higher scores on depression and anxiety scales; and higher BP, plasma BG, TC, and LDL-C levels, but lower HDL-C concentrations. Anxiety symptoms, high education level, and being unmarried were risk factors for SA in patients with MDD with DUIs of <3 months; anxiety symptoms, low BMI, high plasma TC, and low plasma HDL-C were risk factors for SA in the group with DUIs between 3 and 8 months; age, anxiety symptoms, and higher systolic blood pressure were risk factors for those with DUIs > 8 months. This study was a single-center cross-sectional survey, and its limitations include a lack of outside validation. Patients with MDD with and without a SA history have different clinical characteristics and metabolic parameters, and risk factors for suicide vary across DUI stages. Anxiety was a general risk factor, suggesting that clinicians should strengthen their assessment of SA risk in patients with MDD during diagnosis and treatment.

## 1. Introduction

The duration of untreated illness (DUI) refers to the time interval from the first episode of an illness to the correct diagnosis and proper treatment [1, 2]. Many studies have assessed the DUI in patients with psychoses, and an increasing number of reports consider it a potential predictor of disease outcome. Prolonged DUIs may increase the disease burden, lead to a complex disease course and severe symptoms, and affect outcomes [3]. A shortened DUI, in contrast, may prevent disease recurrence or chronicity [4–6]. However, studies on DUIs of different diseases have yielded inconsistent results [7, 8].

The DUI has a broad impact on major depressive disorder (MDD), such as on the effect and response to treatment, severity of symptoms, and disease burden. An earlier study found that patients who receive delayed treatment exhibit greater cognitive impairments than those who receive prompt treatment; the DUI may, thus, be a key factor in the development of cognitive impairment during depressive episodes [9]. Prolonging the DUI can lead to a higher clinical severity of affective spectrum disorders, and the significant association between prolonged DUIs and comorbidities may lead to a worse disease course and adverse outcomes [10]. Shorter DUIs have an overall positive effect on patient responses to treatment as well as remission, which emphasizes the importance of reducing DUIs in populations with depression to prevent worse outcomes and the risk of chronic disease; this is especially important in patients who experience their first episode [11].

Many factors affect DUIs. A study on bipolar disorder (BD) found that the following factors are related to longer DUIs: (1) at least one lifetime marriage/partnership, (2) first psychiatric diagnosis of severe depressive disorder or substance abuse, (3) first episode of depressive polarity, (4) no lifelong psychiatric symptoms, (5) BD type 2, (6) more lifelong depression/mild manic episodes, (7) fewer lifelong manic episodes, (8) suicidal intentions and depressive episodes, and (9) mild manic episodes and hospitalizations. By turns, longer DUI negatively predicted suicide attempts and hospitalizations [12]. A survey of elderly patients with MDD reported that their barriers to seeking help were attributed to stigma, self-motivation, access to formal support, age discrimination, and difficulty in obtaining a preliminary diagnosis. Personal responsibility, mental health literacy, treatment alliances, and informal support are some of the facilitators of help-seeking behaviors [13]. Elderly, less-educated, and married patients with MDD are less likely to seek timely treatment, and those with long DUIs have a higher clinical risk than those with short DUIs [14].

Suicidal attempts (SAs) are a common and complex public health concern worldwide [15], and suicidal ideation and SA are frequent among patients before the disease is effectively treated [16, 17], especially in those with MDD, who are most likely to commit suicide. Depression in patients with first-episode or recent-onset psychosis is associated with high suicidal tendencies, low quality of life, and poor resilience. In populations with first-episode psychosis, depression is related to factors already present during the DUI [18]. Treating patients with MDD who have already attempted suicide is more challenging than caring for those with MDD or a history of SA alone [19, 20]. The presentation of MDD with a history of SA has, therefore, attracted the attention of researchers who attempt to shorten DUIs and provide interventions to high-risk groups as early as possible [21].

However, only few studies have so far reported on the related factors, specifically on risk factors for SA in patients with MDD with different DUIs in the Chinese Han population. Therefore, it is the first study to explore the factors that play a role in SA in MDD and how these factors relate to the DUI in China.

## 2. Materials and Methods

### 2.1. Participants

**Figure 1:**
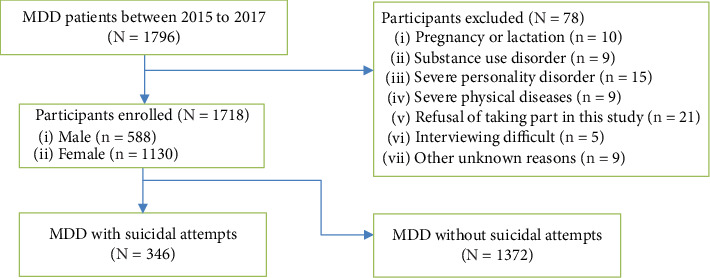
Flow diagram of participant inclusion.

This cross-sectional study was approved by the Ethics Committee of the First Hospital of Shanxi Medical University and was conducted from 2015 to 2017 at the Department of Psychiatry of the First Hospital of Shanxi Medical University. All participants provided informed consent, and their identifiable information was concealed before data collection. The final sample included patients who (1) met the DSM-IV diagnostic criteria for MDD, (2) were aged ≥ 16 but ≤65 years, (3) had no history of prior antidepressant or antipsychotic treatment, (4) scored ≥ 24 points on the 17-item Hamilton depression scale (HAMD), and (5) had at least junior high school education. Exclusion criteria are as follows: (1) having serious physical disease; (2) having neurological disease; (3) having any other major axis I disorder except major depressive disorder; (4) individuals with a history of drug or alcohol abuse/dependence; (5) pregnant or lactating women. A total of 1,796 patients met the inclusion criteria; 78 were excluded for the following reasons: (1) pregnancy or lactation (*n* = 10), (2) substance use disorder (*n* = 9), (3) severe personality disorder (*n* = 15), (4) severe physical diseases (*n* = 9), (5) refusal to participate in the study (*n* = 21), (6) inability to be interviewed due to acute clinical condition (*n* = 5), and (7) other unknown reasons (*n* = 9) (Figure 1). A total of 1,718 patients with MDD (588 male) were thus included. Figure 1 shows the flow diagram of participant inclusion.

### 2.2. Data Collection

All subjects underwent face-to-face interviews and provided demographic and clinical information, including their age, sex, DUI, age of first episode, educational level, marital status, presence or absence of psychotic symptoms (accompanied by hallucinations, delusions, and significant excitatory agitation, psychomotor hysteresis, and catatonia but does not match the diagnosis of schizophrenia), body mass index (BMI), and blood pressure. Depression was assessed using the HAMD-17 [22], which consists of 17 items rated on a 5-point Likert scale ranging from 0 (nonexistent) to 4 (severe). The HAMA-14, which consists of 14 items rated on a 5-point scale from 0 (no symptoms) to 4 (very severe symptoms), was used to evaluate the severity of the patient's anxiety symptoms; the higher the score, the more serious the anxiety symptoms were [23]. The independent blind ratings from the two psychiatrists (both with ≥5 years of clinical experience) who completed the quantitative assessments showed a significant correlation (coefficient: 0.82–0.85). Blood samples from all participants were collected in the morning after fasting for at least 8 hours to measure blood glucose (BG), total cholesterol (TC), triglycerides (TG), low-density lipoprotein cholesterol (LDL-C), and high-density lipoprotein cholesterol (HDL-C) levels. MDD patients who meet one of the following conditions are considered to have metabolic disorders [24]: (1) BMI ≥ 24 kg/m^2^; (2) BG ≥ 6.1 mmol/L; (3) SBP (systolic blood pressure) ≥ 140 mmHg and/or DBP (diastolic blood pressure) ≥ 90 mmHg; (4) TC ≥ 6.2 mmol/L; (5) LDL − C ≥ 4.1 mmol/L; (6) TG ≥ 2.3 mmol/L or HDL − C ≤ 1.0 mmol/L.

### 2.3. Statistical Analysis

All results are expressed as mean ± standard deviation or *n* (%). SPSS 25.0 was used for all statistical analyses. Categorical variables were examined using chi-squared tests. When continuous variables conformed to a normal distribution, they were subjected to analysis of variance (ANOVA) and independent-sample *t*-tests. Nonnormally distributed continuous variables were analyzed using the Mann–Whitney *U* and Wilcoxon rank sum tests. We conducted a univariate analysis to determine differences in demographic information, clinical symptoms, and plasma metabolic parameters between patients with MDD with and without a history of SA. Based on the 25% and 75% DUI quartiles, we then further divided the MDD patients with a SA history into three subgroups: DUI < 3 months, 3 ≤ DUI < 8 months, and DUI ≥ 8 months [14]. Results from multiple comparisons were subjected to Bonferroni correction. Patients at different DUI stages were analyzed using binary logistic regression to explore the risk factors for suicide. Statistical significance was set at *P* < 0.05 (two-tailed).

## 3. Results

### 3.1. Comparison of Clinical Characteristics and Metabolic Parameters between MDD Patients with and without SA History

Of the 1,718 patients with MDD, 346 had MDD complicated with SA, accounting for 20.14% of the sample. MDD patients with a history of SA were older and had longer DUIs than those who had not attempted suicide. The number of patients in the DUI < 3 group who had not attempted suicide was higher than the number of those who had, while the opposite pattern was observed in the DUI ≥ 8 group; there was no significant difference between the groups among patients at the 3 ≤ DUI < 8 stage. MDD patients with SA history were older at disease onset and had higher BP values, higher HAMD and HAMA scores, and higher plasma BG, TC, TG, and LDL-C concentrations, but lower plasma HDL-C concentrations. The proportion of patients with MDD and SA history who were middle school-educated was significantly larger than that of patients who were not; we observed, however, no significant differences in the proportions of patients with other education levels between the two groups. There were no significant differences in sex, marital status, BMI, and metabolic disorders between the two groups (Table 1).

### 3.2. Comparison of Clinical Characteristics and Metabolic Parameters among MDD Patients of Three DUIs

Of all 1718 patients with MDD, 331 patients received their first treatment within 3 months after onset, 895 patients received their first treatment within 3-8 months after onset, and 492 patients received their first treatment eight months after onset. MDD patients with older age, lower educational level, married, higher HAMD score, higher BP, higher plasma BG/TC/LDL-C concentrations, lower plasma HDL-C concentrations, and higher incidence of SA experienced longer DUI. There were no significant differences in gender, psychotic symptoms, HAMA score, BMI, TG, and the incidence of metabolic disorders among different DUI groups (Table 2).

### 3.3. Differences in Clinical Characteristics and Metabolic Parameters between MDD Patients with and without SA History at Different DUIs

Among the 331 MMD patients with a DUI of <3 months, 50 had and 281 had not attempted suicide. The subgroup analysis revealed that in the subgroup with psychotic symptoms, the proportion of patients with MDD and a SA history was higher than that of patients with MDD and no SA history, while the opposite pattern was observed in the absence of psychotic symptoms. Compared with MDD patients with no SA history, those who had attempted suicide had higher BP, higher HAMD and HAMA scores, and higher plasma TC concentrations, but there were no statistically significant differences in plasma BG, TG, LDL-C, and HDL-C concentrations; age; sex; education level; and marital status between the two groups (Table 3).

Among the 895 MDD patients with a DUI ≥3 months but <8 months, 178 had attempted suicide while 717 had not. The patients who had attempted suicide were older; exhibited more psychotic symptoms; had higher BP, HAMD scores, and HAMA scores; and higher plasma BG, TC, and LDL-C concentrations, but lower plasma HDL-C concentrations than the MDD patients with no SA history. There was no significant difference between the two groups in terms of sex, education level, marital status, BMI, and plasma TG concentrations (Table 3).

Of the 492 MDD patients with DUIs ≥ 8 months, 118 had SA history, and 374 did not. Patients who had attempted suicide exhibited more psychotic symptoms and had higher BP, HAMD scores, and HAMA scores, as well as higher plasma BG, TC, and TG concentrations than did MDD patients with no SA history. There were no significant differences between the two subgroups in terms of age, sex, education level, marital status, BMI, and plasma LDL-C and HDL-C concentrations (Table 3).

### 3.4. Related Factors to DUIs in MDD Patients with SA

Pearson correlation analysis was used to analyze the relationship between DUIs in MDD patients with SA and clinical and metabolic indicators. The results showed that the DUIs were positively correlated with age, marital status, and systolic blood pressure and negatively correlated with education (Table 4).

### 3.5. Relationship between Clinical Characteristics and Metabolic Parameters in MDD Patients with SA History at Different DUI Stages

To explore the risk factors for suicide in MDD patients at different DUI stages, we subdivided our sample into three subgroups according to differences in DUI and conducted a binary logistic regression. The results are shown in Table 4–6. The following risk factors were identified for the DUI > 3-month group: educational level, marital status, and HAMA score (Table 5). The following risk factors were identified for the 3–8-month DUI group: HAMA score, BMI, TC, and HDL-C (Table 6). Age, HAMA score, and systolic blood pressure were the risk factors for the DUI > 8 months group (Table 7).

## 4. Discussion

To our knowledge, our study is the first to investigate the relevant clinical, metabolic, and risk factors of SA for MDD patients of Chinese Han ethnicity, with different DUIs and untreated first episodes. Here, we found that (1) the proportion of MDD patients with SA is 20.14%, and MDD patients who experienced DUIs for a longer period of time had the following characteristics: elder, less educated, married, higher HAMD scores, higher BP, higher blood BG/TC/LDL-C concentrations, lower blood HDL-C concentrations, and more likely to attempt suicide; (2) compared with MDD patients without SA, MDD patients with SA have older age, higher blood pressure, higher levels of anxiety and depression, higher blood BG, TC, TG, and LDL-C, lower blood HDL-C concentrations, and more patients with middle school-educated levels; (3) there are differences in clinical characteristics, metabolic parameters, and risk factors for SA in MDD patients among different DUIs.

In an earlier study of patients with treatment-resistant major depression and MDD with SA, the total disease burden was 66.3 thousand disability-adjusted life years (DALYs), while the disease burden of premature death due to suicide was 15.6 thousand DALYs. The same study also found that depression has a negative impact on educational outcomes, marital relationship formation, fertility, and quality of parental care [25]. In our study, the findings are in agreement with those of previous studies [14, 26]. MDD patients with a history of SA were older than those who had not attempted suicide in the 3 ≤ DUI < 8 groups, whereas in the other two subgroups with either shorter or longer DIUs, these differences were not observed. Age at MDD onset was one of the risk factors for SA in the DUI ≥ 8-month subgroup. MDD patients with a history of SA had longer DUIs, and MDD patients with DUI of ≥8 months were more likely to have attempted suicide. Patients with MDD with middle school education were also more likely to have attempted suicide than those with other education levels. In the DUI < 3-month subgroup, high education level and unmarried status were risk factors for SA, which may be related to high self-esteem and a stronger sense of stigma among highly educated people and to the lack of support from marital relationships.

A meta-analysis of studies on patients with MDD with psychotic symptoms found that the presence of symptoms was an important risk factor for suicide in depressed patients, with the risk being increased not just during the acute phase of the disease but throughout the patient's life [27]. The results of a 16-year longitudinal study suggest that clinicians should improve the identification of comorbid anxiety in MDD because anxiety symptoms in patients with MDD also increase the risk of suicide [28]. Patients with MDD and severe depression are likely to commit suicide [29]. Our results, which are overall consistent with those of previous studies, show that a higher HAMA score was the only cross-subgroup risk factor in our sample, that is, the presence of anxiety symptoms at any DUI stage was a risk factor for SA in our patients with MDD.

A previous study found that the variability of metabolic parameters was an independent predictor of MDD [30], and Marijnissen et al. found that metabolic disorders indicated a poor course of MDD later in life [31]. Other studies have reported conflicting results [32, 33] that may stem from the fact that MDD is a multifactorial disease with highly heterogeneous symptoms [34]. Herein, we examined the differences in metabolic parameters of MDD patients with and without a history of SA, and our results showed that the BP and BG, TC, TG, and LDL-C concentrations were higher in patients who had attempted than in those who had not attempted suicide, while HDL-C levels were lower in patients without a history of SA. However, the findings on metabolic parameters were inconsistent across our different subgroups. In the DUI < 3-month group, BP and TC concentrations were higher in patients who had attempted suicide, but no other indicators differed between the SA history subgroups. In the 3 ≤ DUI < 8-month group, BP and BG, TC, and LDL-C concentrations were higher, whereas HDL-C levels were lower in patients who had attempted suicide. In the DUI ≥ 8-month group, there was no difference between the groups with and without a history of SA in LDL-C and HDL-C levels, while all other metabolic parameters were higher in patients with a history of SA. Overall, BMI, TC, and HDL-C were risk factors for SA at 3 ≤ DUI < 8 months, and systolic blood pressure was a risk factor at DUI ≥ 8 months.

It is worth noting that the incidence of metabolic disorders in this study did not differ between MDD with and without SA, nor between different DUIs, but the mean value was significantly different, which suggested that clinicians should pay attention to elevated but not abnormal metabolic markers, especially higher systolic blood pressure indicating a higher risk of SA. At the same time, further studies should be designed to explore the cutoff value of metabolic indexes in MDD with SA patients.

This study has some limitations. First, this was a single-center cross-sectional survey; therefore, the generalizability of our results might be limited. Second, patient status was only assessed during outpatient visits; interviews with family members or spouses were not conducted.

## 5. Conclusions

We found that patients with MDD with psychotic symptoms and high anxiety scores were more likely to commit suicide and that the metabolic parameters of patients with MDD with a history of SA differed across different DUI stages. In the early stage of MDD (DUI < 3 months), high HAMA scores, high education level, and unmarried status were risk factors for SA in our sample. In patients with MDD who were not treated for 3–8 months after onset, high HAMA scores, low BMI, high TC concentrations, and low HDL-C levels were the risk factors for SA. Among those who were not treated for MDD for more than 8 months, high HAMA scores, high systolic blood pressure, and younger age were risk factors for SA. In conclusion, the study suggests that (1) the longer the DUIs, the higher the risk MDD patients are facing. Clinicians should improve their ability to recognize MDD and shorten DUIs. (2) When receiving MDD patients, clinicians should pay attention to their DUIs, evaluate their risk factors, and prevent the occurrence of SAs.

## Figures and Tables

**Table 1:** Comparison of clinical characteristics and metabolic parameters between MDD patients with and without a history of SA (*M* ± *S*/*n* [%]).

	MDD with SA (*n* = 346)	MDD without SA (*n* = 1372)	*χ* ^2^/*t*	*P* value
Age (years)	36.12 ± 12.35	34.55 ± 12.43	2.27	0.04
Gender (*n*/%)				
Male (*n* = 588)	112 (32.37)	476 (34.69)	0.66^△^	0.45
Female (*n* = 1130)	234 (67.63)	896 (65.31)		
DUI (months)	6.95 ± 4.89	6.15 ± 4.68	2.83	0.01
Subgroups				
DUI < 3 (*n* = 331)	50 (14.45)	281 (20.48)	9.77^△^	<0.01
3 ≤ DUI < 8 (*n* = 895)	178 (51.45)	717 (52.26)		
DUI ≥ 8 (*n* = 492)	118 (34.10)	374 (27.26)		
Educational level (n/%)				
Middle school (*n* = 413)	100 (28.90)	313 (22.81)	8.09^△^	0.04
High school (*n* = 760)	141 (40.75)	619 (45.12)		
College/university (*n* = 449)	81 (23.41)	368 (26.82)		
Postgraduate education (*n* = 96)	24 (6.94)	72 (5.25)		
Marital status (*n*/%)				
Single (*n* = 502)	95 (27.46)	407 (29.66)	0.65^△^	0.43
Married (*n* = 1216)	251 (72.54)	965 (70.34)		
Psychotic symptom (*n*/%)				
Yes (*n* = 171)	88 (25.43)	83 (6.05)	1115.84^△^	<0.01
No (*n* = 1547)	258 (74.57)	1289 (93.95)		
HAMD	32.24 ± 2.89	29.81 ± 2.75	14.57	<0.01
HAMA	23.59 ± 3.54	20.09 ± 3.08	16.83	<0.01
BMI (kg/m^2^)	24.32 ± 2.33	24.38 ± 1.81	0.24	0.81
BP (mmHg)				
Systolic blood pressure	124.40 ± 11.97	118.24 ± 10.27	8.60	<0.01
Diastolic blood pressure	78.56 ± 7.69	75.29 ± 6.31	7.10	<0.01
BG (mmol/L)	5.59 ± 0.74	5.35 ± 0.61	5.81	<0.01
TC (mmol/L)	5.77 ± 1.11	5.11 ± 1.07	9.54	<0.01
TG (mmol/L)	2.29 ± 1.01	2.14 ± 0.98	2.89	<0.01
LDL-C (mmol/L)	3.21 ± 0.91	2.93 ± 0.84	5.55	<0.01
HDL-C (mmol/L)	1.14 ± 0.29	1.24 ± 0.28	6.37	<0.01
Metabolic disorders				
With (*n* = 1441)	301 (86.99)	1140 (83.09)	3.11^△^	0.08
Without (*n* = 277)	45 (13.01)	232 (16.91)		

^△^The *χ*^2^ value. Abbreviations: SA: suicide attempt; DUI: duration of untreated illness; HAMD: 17-item Hamilton depression scale; HAMA: 14-item Hamilton anxiety scale; BMI: body mass index; BP: blood pressure; BG: blood glucose; TC: total cholesterol; TG: triglycerides; LDL-C: low-density lipoprotein cholesterol; HDL-C: high-density lipoprotein cholesterol.

**Table 2:** Differences of clinical characteristics and metabolic parameters of MDD patients in different DUI stages (*M* ± *S*/*n* [%]).

	DUI < 3 (*n* = 331)	3 ≤ DUI < 8 (*n* = 895)	DUI ≥ 8 (*n* = 492)	*χ* ^2^/*F*	*P* value
Age (years)	30.66 ± 12.52	33.45 ± 12.00	39.56 ± 11.18	65.33	<0.01
Gender (*n*/%)					
Male (*n* = 588)	109 (32.90)	324 (36.20)	155 (31.50)	3.42^△^	0.18
Female (*n* = 1130)	222 (67.10)	571 (63.80)	337 (68.50)		
Educational level (*n*/%)					
Middle school (*n* = 413)	53 (16.00)	181 (20.20)	179 (36.40)	69.96^△^	<0.01
High school (*n* = 760)	176 (53.20)	389 (43.50)	195 (39.60)		
College/university (*n* = 449)	87 (26.30)	266 (29.70)	96 (19.50)		
Postgraduate education (*n* = 96)	15 (4.50)	59 (6.60)	22 (4.50)		
Marital status (n/%)					
Single (*n* = 502)	144 (43.500)	279 (31.20)	79 (16.10)	75.53^△^	<0.01
Married (*n* = 1216)	187 (56.50)	616 (68.80)	413 (83.90)		
Psychotic symptom (n/%)					
Yes (*n* = 171)	28 (8.50)	89 (9.90)	54 (11.00)	1.40^△^	0.50
No (*n* = 1547)	303 (91.50)	806 (90.10)	438 (89.00)		
HAMD	29.81 ± 2.86	30.26 ± 2.99	30.69 ± 2.86	9.05	<0.01
HAMA	20.43 ± 3.17	20.82 ± 3.52	20.99 ± 3.56	2.65	0.07
BMI (kg/m^2^)	24.17 ± 1.83	24.37 ± 1.99	24.50 ± 1.85	2.73	0.07
BP (mmHg)					
Systolic blood pressure	115.63 ± 10.78	118.87 ± 10.70	123.19 ± 10.28	53.51	<0.01
Diastolic blood pressure	74.70 ± 6.58	75.79 ± 6.60	77.07 ± 6.95	12.87	<0.01
BG (mmol/L)	5.32 ± 0.65	5.40 ± 0.66	5.46 ± 0.61	4.88	<0.01
TC (mmol/L)	5.03 ± 1.08	5.23 ± 1.12	5.43 ± 1.07	13.36	<0.01
TG (mmol/L)	2.12 ± 0.96	2.19 ± 1.00	2.16 ± 0.97	0.63	0.53
LDL-C (mmol/L)	2.89 ± 0.86	2.95 ± 0.85	3.11 ± 0.86	7.89	<0.01
HDL-C (mmol/L)	1.25 ± 0.26	1.23 ± 0.29	1.18 ± 0.30	4.85	0.01
Suicide attempts (*n*)					
With (*n* = 346)	50 (15.10)	178 (19.90)	118 (24.00)	9.77^△^	0.01
Without (*n* = 1372)	281 (84.90)	717 (80.10)	374 (76.00)		
Metabolic disorders					
With (*n* = 1441)	265 (80.10)	754 (84.20)	422 (85.80)	4.96^△^	0.08
Without (*n* = 277)	66 (19.90)	141 (15.80)	70 (14.20)		

^△^The *χ*^2^ value. Abbreviations: DUI: duration of untreated illness; HAMD: 17-item Hamilton depression scale; HAMA: 14-item Hamilton anxiety scale; BMI: body mass index; BP: blood pressure; BG: blood glucose; TC: total cholesterol; TG: triglycerides; LDL-C: low-density lipoprotein cholesterol; HDL-C: high-density lipoprotein cholesterol.

**Table 3:** Differences of clinical characteristics and metabolic parameters of MDD patients with and without SA in different DUI stages (*M* ± *S*/*n* [%]).

	DUI < 3 (*n* = 331)				3 ≤ DUI < 8 (*n* = 895)				DUI ≥ 8 (*n* = 492)			
	MDD with SA (*n* = 50)	MDD without SA (*n* = 281)	*χ* ^2^/*t*	*P* value	MDD with SA (*n* = 178)	MDD without SA (*n* = 717)	*χ* ^2^/*t*	*P* value	MDD with SA (*n* = 118)	MDD without SA (*n* = 374)	*χ* ^2^/*t*	*P* value
Age (years)	30.08 ± 11.94	30.77 ± 12.64	0.36	0.72	36.63 ± 12.28	32.90 ± 11.87	2.72	<0.01	39.42 ± 11.63	40.56 ± 11.19	0.96	0.34
Gender (*n*/%)												
Male	15 (30.00)	94 (33.50)	0.23^△^	0.74	61 (34.30)	263 (36.70)	0.36^△^	0.60	36 (30.50)	119 (31.80)	0.07^△^	0.82
Female	35 (70.00)	187 (66.50)			117 (65.70)	454 (63.30)			82 (69.50)	255 (68.20)		
Educational level (*n*/%)												
Middle school	7 (14.00)	46 (16.40)	2.33^△^	0.51	45 (25.30)	136 (19.00)	4.03^△^	0.26	48 40.70)	131 (35.00)	6.09^△^	0.11
High school	23 (46.00)	153 (54.40)			75 (42.10)	314 (43.8)			43 (36.40)	152 (40.60)		
College/university	17 (34.00)	70 (24.90)			46 (25.80)	220 (30.70)			18 (15.30)	78 (20.90)		
Postgraduate education	3 (6.00)	12 (4.30)			12 (6.70)	47 (6.60)			9 (7.60)	13 (3.50)		
Marital status (*n*/%)												
Single	27 (54.00)	117 (41.60)	2.64^△^	0.12	49 (27.50)	230 (32.10)	1.38^△^	0.28	19 (16.10)	60 (16.00)	0.00^△^	1.00
Married	23 (46.00)	164 (58.40)			129 (72.50)	487 (67.90)			99 (83.90)	314 (84.00)		
Psychotic symptom (*n*/%)												
Yes	12 (24.00)	16 (5.70)	18.37^△^	<0.01	46 (25.80)	43 (6.00)	62.71^△^	<0.01	30 (25.40)	24 (6.40)	33.16^△^	<0.01
No	38 (76.00)	265 (94.30)			132 (74.20)	674 (94.00)			88 (74.60)	350 (93.60)		
HAMD	31.72 ± 2.94	29.47 ± 2.71	5.33	<0.01	32.43 ± 2.92	29.72 ± 2.76	11.57	<0.01	32.18 ± 2.81	30.22 ± 2.71	6.77	<0.01
HAMA	23.00 ± 3.01	19.98 ± 2.98	6.60	<0.01	23.87 ± 3.72	20.07 ± 3.04	12.65	<0.01	23.42 ± 3.47	20.23 ± 3.23	9.17	<0.01
BMI (kg/m^2^)	24.54 ± 1.82	24.11 ± 1.82	1.51	0.13	24.18 ± 2.47	24.41 ± 1.86	1.19	0.24	24.45 ± 2.29	24.51 ± 1.69	0.29	0.77
BP (mmHg)												
Systolic blood pressure	119.64 ± 11.39	114.92 ± 10.53	2.89	<0.01	124.13 ± 11.97	117.56 ± 9.94	6.77	<0.01	126.82 ± 11.66	122.05 ± 9.53	4.04	<0.01
Diastolic blood pressure	76.76 ± 6.26	74.34 ± 6.57	2.42	0.02	78.58 ± 8.00	75.10 ± 6.01	5.43	<0.01	79.31 ± 7.70	76.37 ± 6.54	3.74	<0.01
BG (mmol/L)	5.48 ± 0.64	5.29 ± 0.65	1.92	0.06	5.59 ± 0.78	5.34 ± 0.61	3.94	<0.01	5.63 ± 0.72	5.41 ± 0.56	2.98	<0.01
TC (mmol/L)	5.52 ± 1.21	4.94 ± 1.03	3.59	<0.01	5.85 ± 1.13	5.08 ± 1.06	8.22	<0.01	5.77 ± 1.02	5.32 ± 1.07	4.06	<0.01
TG (mmol/L)	2.18 ± 0.98	2.10 ± 0.96	0.54	0.59	2.29 ± 0.99	2.16 ± 1.00	1.49	0.14	2.35 ± 1.07	2.11 ± 0.93	2.38	0.02
LDL-C (mmol/L)	3.10 ± 1.06	2.85 ± 0.82	1.62	0.11	3.23 ± 0.91	2.88 ± 0.83	4.99	<0.01	3.22 ± 0.84	3.07 ± 0.87	1.62	0.11
HDL-C (mmol/L)	1.18 ± 0.28	1.26 ± 0.25	1.89	0.06	1.12 ± 0.29	1.26 ± 0.29	5.76	<0.01	1.14 ± 0.30	1.20 ± 0.30	1.66	0.10

^△^The *χ*^2^ value. Abbreviations: DUI: duration of untreated illness; SA: suicide attempt; HAMD: 17-item Hamilton depression scale; HAMA: 14-item Hamilton anxiety scale; BMI: body mass index; BP: blood pressure; BG: blood glucose; TC: total cholesterol; TG: triglycerides; LDL-C: low-density lipoprotein cholesterol; HDL-C: high-density lipoprotein cholesterol.

**Table 4:** Related factors to DUIs in MDD patients with SA.

	*r*	*P* value
Age (years)	0.23	<0.01
Educational level	-0.15	0.01
Marital status	0.27	0.01
Systolic blood pressure	0.17	0.01

**Table 5:** Factors associated with SA in MDD patients with DUI < 3 months.

	Coefficients	Std. error	Wald	*P* value	95% confidence interval for exp (B)		
	B				Exp (B)	Lower	Upper
Constant	-20.46	4.66	19.29	<0.01	0.00		
Educational level	0.73	0.27	7.70	0.01	2.08	1.24	3.50
Marital status	-1.32	0.49	7.31	0.01	0.27	0.10	0.70
HAMA	0.29	0.08	12.22	<0.01	1.33	1.14	1.58

Abbreviations: HAMA: 14-item Hamilton anxiety scale.

**Table 6:** Factors associated with SA in MDD patients with 3 ≤ DUI < 8.

	Coefficients	Std. error	Wald	*P* value	95% confidence interval for exp (B)		
	B				Exp (B)	Lower	Upper
Constant	-11.61	2.20	27.87	<0.01	0.00		
HAMA	0.27	0.04	45.31	<0.01	1.31	1.21	1.42
BMI	-0.13	0.05	6.66	0.01	0.88	0.79	0.97
TC	0.31	0.12	6.23	0.01	1.36	1.07	1.73
HDL-C	-0.89	0.35	6.43	0.01	0.41	0.21	0.82

Abbreviations: HAMA: 14-item Hamilton anxiety scale; BMI: body mass index; TC: total cholesterol; HDL-C: high-density lipoprotein cholesterol.

**Table 7:** Factors associated with SA in MDD patients with DUI ≥ 8 months.

	Coefficients	Std. error	Wald	*P* value	95% confidence interval for exp (B)		
	B				Exp (B)	Lower	Upper
Constant	-11.95	2.91	16.90	<0.01	0.00		
Age	-0.06	0.02	12.70	<0.01	0.94	0.91	0.97
HAMA	0.24	0.05	23.57	<0.01	1.27	1.16	1.41
Systolic blood pressure	0.04	0.02	5.24	0.02	1.04	1.01	1.08

Abbreviations: HAMA: 14-item Hamilton anxiety scale.

## Data Availability

The data that support the findings of this study are available from the corresponding author, Zhang Xiangyang, upon reasonable request.
